# Effect of Gold Nanoparticles and Silicon on the Bioactivity and Antibacterial Properties of Hydroxyapatite/Chitosan/Tricalcium Phosphate-Based Biomicroconcretes

**DOI:** 10.3390/ma14143854

**Published:** 2021-07-09

**Authors:** Joanna Czechowska, Ewelina Cichoń, Anna Belcarz, Anna Ślósarczyk, Aneta Zima

**Affiliations:** 1 Department of Ceramics and Refractories, Faculty of Materials Science and Ceramics, AGH University of Science and Technology, Mickiewicza 30 Av., 30-059 Krakow, Poland; ecichon@agh.edu.pl (E.C.); aslosar@agh.edu.pl (A.Ś.); 2 Chair and Department of Biochemistry and Biotechnology, Medical University in Lublin, Chodzki 1, 20-093 Lublin, Poland; annabelcarz@umlub.pl

**Keywords:** biomicroconcretes, gold nanoparticles, silicon

## Abstract

Bioactive, chemically bonded bone substitutes with antibacterial properties are highly recommended for medical applications. In this study, biomicroconcretes, composed of silicon modified (Si-αTCP) or non-modified α-tricalcium phosphate (αTCP), as well as hybrid hydroxyapatite/chitosan granules non-modified and modified with gold nanoparticles (AuNPs), were designed. The developed biomicroconcretes were supposed to combine the dual functions of antibacterial activity and bone defect repair. The chemical and phase composition, microstructure, setting times, mechanical strength, and in vitro bioactive potential of the composites were examined. Furthermore, on the basis of the American Association of Textile Chemists and Colorists test (AATCC 100), adapted for chemically bonded materials, the antibacterial activity of the biomicroconcretes against *S. epidermidis*, *E. coli*, and *S. aureus* was evaluated. All biomicroconcretes were surgically handy and revealed good adhesion between the hybrid granules and calcium phosphate-based matrix. Furthermore, they possessed acceptable setting times and mechanical properties. It has been stated that materials containing AuNPs set faster and possess a slightly higher compressive strength (3.4 ± 0.7 MPa). The modification of αTCP with silicon led to a favorable decrease of the final setting time to 10 min. Furthermore, it has been shown that materials modified with AuNPs and silicon possessed an enhanced bioactivity. The antibacterial properties of all of the developed biomicroconcretes against the tested bacterial strains due to the presence of both chitosan and Au were confirmed. The material modified simultaneously with AuNPs and silicon seems to be the most promising candidate for further biological studies.

## 1. Introduction

Bioactivity, appropriate mechanical strength, ease of handling, and antibacterial properties are of paramount importance for the commercial success of bone grafts [1]. From a medical point of view, chemically bonded materials in the form of shapeable pastes are a very attractive alternative for pre-shaped sintered bioceramics. One of the most widely investigated groups of these biomaterials are calcium phosphate-based bone cements (CPCs). These chemically bonded bone substitutes exhibit both biocompatibility and high surgical handiness [2,3,4,5]. In particular, CPCs enriched with microbeads, granules, and microspheres have gained huge interest in recent years. Polymeric microbeads (e.g., alginate, hyaluronic acid, and PLGA—poly(lactic-co-glycolic acid)) as well as hybrid or inorganic granules (e.g., bioglass and calcium sulfate dihydrate) were introduced into the CPCs matrix in order to obtain biomicroconcretes [6,7,8,9]. The produced composites possessed some advantages such as an improved bioactivity, injectability, washout resistance, and mechanical properties [10,11,12,13]. Furthermore, applied granules, microspheres, and microbeads, when enriched with drug and/or biologically active agents, can serve as delivery vehicles in local drug delivery systems or targeted therapies. As bone defect-related surgical procedures carry potential inflammation and infection risks, antibacterial drugs or compounds containing elements such as silver, copper, and gold are often introduced into biomaterials [14,15,16,17]. Inorganic-organic hybrids with antibacterial nanoparticles (NPs) or polymers are also intensively studied, and seem to be promising candidates for medical applications [18,19,20]. Metal-based nanoparticles are known to have non-specific bacterial toxicity mechanisms. This phenomenon broadens the spectrum of antibacterial activity and makes the development of resistance by bacteria difficult [21]. As a result, a large majority of metal-based nanoparticles such as AgNPs, AuNPs, or CuNPs act effectively on both Gram-positive and Gram-negative bacteria. According to the literature, the antimicrobial mechanism of action of NPs is generally described by the following three models: (I) oxidative stress induction, (II) metal ion release, and (III) non-oxidative mechanisms [22]. They can also occur simultaneously.

In order to obtain bone substitutes with an improved biological performance, calcium phosphates (CaPs) modified with various chemical elements such as silicon, magnesium, zinc, or strontium have been recently developed [23,24,25]. It is well known that silicon (Si) serves as a beneficial bioactive element, and a higher in vitro and in vivo bioactivity of silicon modified bioceramics has been already reported in the literature [26,27]. The presence of silicon is supposed to stimulate osteogenesis, thus resulting in bone repair and regeneration with fully functional tissue. It has been shown that both Si-HA- (Si-doped hydroxyapatite) and Si-TCP (Si-doped tricalcium phosphate)-based materials exhibit enhanced bone apposition, bone in-growth, and cell-mediated degradation in comparison with their non-modified counterparts [28,29].

In the present study, we proposed a complex inorganic-organic system combining the bioactivity of surgically handy CPCs and the antibacterial properties of hybrid AuNPs/hydroxyapatite/chitosan granules. Because of reports of a high bioactivity of Si-doped bioceramics, silicon-modified αTCP was synthesized and used in our study. We assumed that the introduction of silicon and gold nanoparticles into the biomicroconcretes may have a beneficial impact on their physicochemical and biological properties. The effect of these modifiers on the bioactive potential and antibacterial activity of biomicroconcretes against *Staphylococcus epidermidis*, *Escherichia coli*, and *Staphylococcus aureus* was examined.

## 2. Materials

### 2.1. Hybrid Hydroxyapatite-Chitosan Granules (HA/CTS)

Hybrid hydroxyapatite (HA)/chitosan (CTS) granules (HA/CTS) containing 17 wt.% chitosan were synthesized via a wet chemical method according to the procedure previously described by Zima [20]. Briefly, phosphoric acid (Eurochem BGD, Tarnow, Poland) was introduced directly to the chitosan solutions in acetic acid(CH_3_COOH, POCH, Gliwice, Poland), and the obtained mixtures were added dropwise to the Ca(OH)_2_ suspension. Medium molecular weight chitosan (around 100,000 kDa, deacetylation degree ≥75.0%, viscosity 200–800 CPS, Sigma-Aldrich, St. Louis, MO, USA), Ca(OH)_2_ (Merck, Darmstadt, Germany), and H_3_PO_4_ (85.0 wt.%, Eurochem BGD, Tarnow, Poland) were applied. The suspension was aged for 24 h and then decanted. The precipitate was washed with distilled water, centrifuged, and frozen (48 h). After defrosting, the obtained filter cakes were sieved and dried to obtain hybrid granules.

### 2.2. Hybrid HA/CTS Granules Modified with Gold Nanoparticles (AuNPs-HA/CTS)

Gold modified HA/CTS granules containing 17 wt.% chitosan (AuNPs-HA/CTS) were also obtained via a wet chemical synthesis performed similarly. Gold was introduced in the amount of 0.1 wt.%. The gold nanoparticles (AuNPs; 99.99% Au, APS-14 nm, US Research Nanomaterials, Houston, TX, USA) were added to the HA/CTS precipitate before frizzing step. In this case, after thawing, self-assembling granules were obtained. The AuNPs-HA/CTS granules were then dried and sieved to divide them into appropriate fractions.

### 2.3. α-Tricalcium Phosphate and Silicon Modified αTCP Powders

The α-tricalcium phosphate (αTCP) powder was synthesized by the wet chemical method, using chemically pure grade Ca(OH)_2_ (POCH, Gliwice, Poland) and an 85 wt.% solution of H_3_PO_4_ (POCH, Gliwice, Poland) as the substrates. In the case of Si modified αTCP (Si-αTCP), as the source of silicon, the silicon tetraacetate (Sigma Aldrich, Poznan, Poland) was used. Silicon was introduced in the amount of 0.3 wt.%. The obtained precipitates were dried, sintered (1300 °C), ground in an attritor, and sieved (<0.063 mm).

### 2.4. Hydroxyapatite/Chitosan/Tricalcium Phosphate-Based Biomicroconcretes

The initial solid phases of the HT, Au-HT, and Au, Si-HT materials were obtained by mixing the hybrid granules (H) (300–400 µm) with α-tricalcium phosphate powders (T) in a ratio of 2:3, respectively. In order to ensure the homogeneity of the composites, the biomicroconcretes were prepared by two-step hand mixing. In the first step, the αTCP powder was mixed with the granules using a spatula for 1 min, to ensure homogenous distribution of the solid phase constituents. Afterward, the liquid phase was added to the solid phase and the components were stirred for about 30–40 s to produce a moldable, setting, and hardening paste. The 0.75 wt.% methylcellulose solution in 2.0 wt.% solution of Na_2_HPO_4_ was applied as the liquid phase of the biomicroconcretes (Table 1). Samples for measurements were prepared by mixing the components in a liquid to powder ratio (L/P) equal 0.6 g/g. In order to produce samples of a desired shape and size, the obtained modulable pastes were introduced onto Teflon molds and left to set.

## 3. Methods

### 3.1. Chemical and Phase Composition

The X-ray fluorescence method (XRF) was applied to check the chemical composition of the initial powders and granules (WDXRF Axios Max, PANalytical, Malvern, UK). The phase compositions of the studied materials were characterized using the X-ray diffraction method (D2 Phaser diffractometer, Bruker, Billerica, MA, USA) using CuKα radiation (1.54 Å). The intensity of the reflexes was recorded in a 2θ range, from 10° to 90° at 0.02° intervals. The crystalline phases were identified by comparing the experimental diffractograms to the International Centre for Diffraction Data (ICDD) Standards: hydroxyapatite (01-076-0694) and α-tricalcium phosphate (00-009-0348). Phase quantification was performed according to the Rietveld refinement method using (Diffrac.Suite TOPAS Rietveld Analysis Software, (Version 4.2.0.1, 2011, Bruker, Billerica, MA, USA). All of the measurements were done in triplicate and the results are presented as the mean ± standard deviation (SD).

### 3.2. Setting Times

The setting times of the biomicroconcretes were identified according to the ASTM C266-20 standard using Gilmore Needles (Humbold MFG Co., Norridge, IL, USA) [30]. The Gillmore Apparatus has two steel weights needles. The initial setting needle has a diameter of 2.12 mm and a weight of 113 g. The final setting needle has a diameter of 1.06 mm and a weight of 453.6 g. In order to measure the setting times, the biomicroconcrete was placed in a special form (8 mm × 10 mm × 5 mm) and the needle of the apparatus was lightly applied to its surface. The setting time is a penetration measurement that does not mark the surface with a complete circular sign. All of the experiments were performed at room temperature (23 ± 2 °C). The results are presented as the average value of three measurements ± standard deviations (SD).

### 3.3. Compressive Strength

The compressive strength measurements were done on cylindrical samples (12 mm in height and 6 mm in diameter) using a universal material testing machine (Instron 3345, Norwood, MA, USA) at a crosshead displacement rate of 1 mm·min^−1^. The results of the compressive strength are expressed as the mean ± SD error of the then-fold determinations. A one-way ANOVA followed by post hoc Tukey’s Honest Significant Difference (HSD) were applied to check if the differences between the materials were statistically significant (*p* < 0.01).

### 3.4. Microstructure

The microstructures of the biomicroconcretes, the granule/matrix interfaces, and the surfaces after the incubation of materials in simulated body fluid were observed via scanning electron microscope (Nova 200 NanoSEM, FEI Company, Hillsboro, OR, USA). The energy dispersive spectrometer (EDS) was used to examine the chemical compositions in the micro-areas. The accelerating voltage was equal to 18 kV. Before examination, the surfaces of all of the samples were covered with a thin carbon film. Transmission electron microscopy (TEM) investigations were performed using a FEI TECNAI G2 200 kV FEG transmission electron microscope (FEI Company, Hillsboro, OR, USA), equipped with side entry for bright field (BF) and selected area (electron) diffraction (SAED) and bottom-entry for high-resolution episcopic microscopy (HREM) imaging cameras.

### 3.5. Chemical Stability and Bioactivity In Vitro

The chemical stability and bioactivity were evaluated after incubating materials in standard simulated body fluid (SBF) prepared according to the Kokubo’s protocol [31]. Briefly, the cylindrical samples were placed into the containers (FL Medical, Torreglia (Padova), Italy) with SBF and stored at 37 °C. The chemical stability of biomicroconcretes was tested by measuring pH vs. time of immersion of the samples in SBF. pH measurements were taken using pH/conduct-meter Hanna H198129 Combo (Hannah Instruments, Woonsocket, RI, USA). Each measurement was repeated three times. Afterward, samples were removed from SBF, rinsed with distilled water and dried at temperatures below 40 °C. To confirm the bioactive potential SEM observations of the biomicroconcretes surfaces were done. Experimental data were processed by Excel Microsoft 2019 (Version: 1808, Microsoft Corp.—Redmond, WA, USA) and the results were presented as mean ± S.D.

### 3.6. In Vitro Antibacterial Activity

#### 3.6.1. Microorganisms and Culture Conditions

Reference Gram-positive (*Staphylococcus aureus* ATCC 25923 and *Staphylococcus epidermidis* ATCC 12228) and Gram-negative (*Escherichia coli* ATCC 25922) bacterial strains were used in antibacterial activity tests. The strains were maintained in microbanks (Technical Service Consultants Limited, Heywood, UK) at −80 °C as sterile stocks. Directly before the tests, bacteria were transplanted onto fresh slant Mueller-Hinton (M-H, Biomaxima, Lublin, Poland) agar medium and incubated at 37 °C for 24 h. To obtained starting bacterial suspension, a single bacterial colony was transferred into the Mueller-Hinton broth and incubated for 24 h at 37 °C. Then, propagated bacteria were then washed 3 times in sterile 0.9% NaCl (POCH, Gliwice, Poland) followed by centrifugation (10 min, 2000 rpm) and suspended in M-H broth diluted 250 times in sterile 0.9% NaCl solution (to obtain the suspension promoting bacterial survival but not propagation). The starting suspension was prepared directly before the experiments and then diluted to obtain a working suspension.

#### 3.6.2. Antibacterial Activity Test

The evaluation of the antibacterial activity of tested samples was performed on basis of AATCC Test Method 100-2019 “Antibacterial finishes on textile materials: assessment of.”, adapted for chemically bonded materials. Briefly, cylindrical samples were first sterilized by ethylene oxide method in a paper/plastic peel pouch (1 h, 55 °C, followed by aeration for 20 h). Working bacterial suspension (1.5 × 10^5^ CFU/mL) of each bacterial strain was prepared from starting suspensions using M-H broth diluted 250 times in sterile 0.9% NaCl. Samples of control HT material and tested Au-HT and Au, Si-HT biomicroconcretes were placed on sterile Petri dishes (in quadruplicate). 45 µL of working bacterial suspension (volume which was completely absorbed by biomicroconcretes samples without the excess leaching outside) was carefully placed onto each sample, allowed to soak into the material and then incubated at 37 °C for 24 h. Afterward, the samples (T_24h_), were transferred into 4.5 of sterile 0.9% NaCl and vigorously shaken for 60 s to elute the bacterial cells which were then plated onto M-H agar using EasySpiral Dilute (Interscience, Saint Nom la Brétèche, France) automatic plater (each sample in triplicate). Another set of control samples was treated as above without immediately after absorbing the inoculate (T_0h_), without incubation. M-H agar plates with plated bacteria eluted from samples were incubated at 37 °C for 48 h. CFU were then counted for each plate. Percent reduction of bacteria due to Au and Si introduction to the cements was calculated by the Equation (1):

% reduction = [(B − A)B] × 100(1)

where:

B—the number of bacteria recovered from the inoculated control specimen immediately after the inoculation (T_0h_)

A—the number of bacteria recovered from the inoculated treated (modified) specimen 24 h after the inoculation (T_24h_).

#### 3.6.3. Statistics

Two statistical analyses were performed—for evaluation of the effect of chitosan presence in non-modified biomicroconcrete (HT) and for evaluation of AuNPs- and Si-modified materials. The Mann–Whitney test (chosen because of non-normal distribution in some groups) was used to assess the statistical differences (*p* < 0.05) between biomicroconcretes incubated with bacteria for 24 h and not incubated with bacteria (0 h). A one-way ANOVA test followed by Dunnett’s test was selected for the evaluation of the statistical differences between AuNPs- and AuNPs/Si-modified materials and HT (24 h) (*p* < 0.05). All of the results were presented as mean ± SD. Experimental data were processed by Excel Microsoft 2019 (Version: 1808, Microsoft Corp., Redmond, WA, United States) and GraphPad Prism 5, Version 5.03 software (2009, GraphPad Software, San Diego, CA, USA).

## 4. Results and Discussion

### 4.1. Chemical and Phase Composition

**Figure 1:**
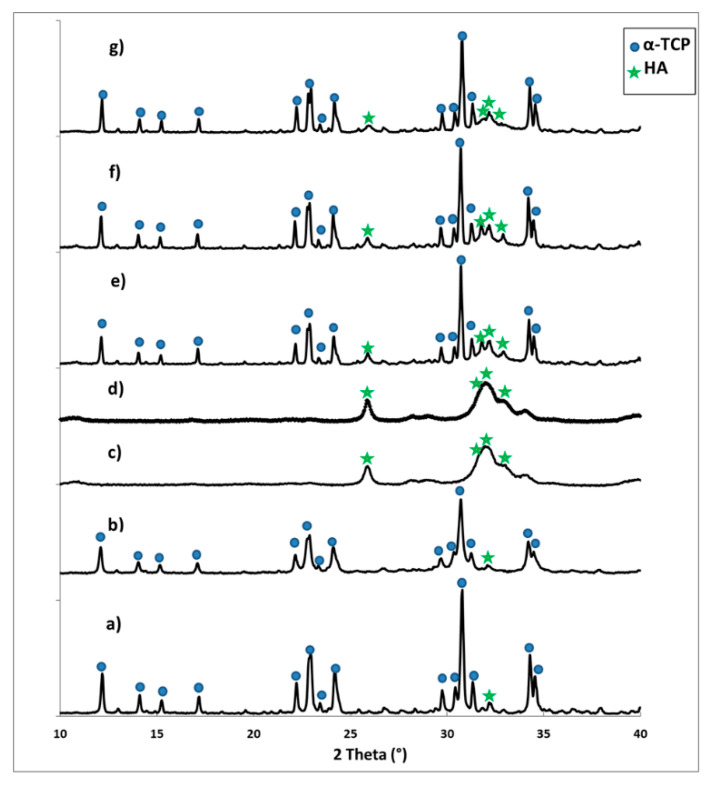
XRD patterns of materials: (**a**) αTCP (αTricalcium Phosphate) powder, (**b**) Si-αTCP (silicon-doped αTricalcium Phosphate) powder, (**c**) hybrid hydroxyapatite/chitosan granules, (**d**) hybrid hydroxyapatite/chitosan granules containing gold nanoparticles, (**e**) material HT, (**f**) material Au-HT, and (**g**) material Au, Si-HT.

The X-ray fluorescence method confirmed the presence of 0.17 wt.% gold in the AuNPs-HA/CTS granules and 0.26 wt.% of silicon in the initial Si-αTCP powder. The XRD analysis revealed that the initial α-tricalcium phosphate and silicon modified the Si-α-tricalcium phosphate (Si-αTCP) powders composed mainly of αTCP (97–98 wt.%) and a small amount of hydroxyapatite phase (2–3 wt.%) (Figure 1a,b and Table 2). According to Reid et al. [32], the amount of silicon influences the phase composition of the final materials and the limit of silicon substitution to αTCP is approximately 1.8 wt.%. However, not only the amount of silicon, but also its source and the method of synthesis is relevant. Szurkowaska et al. [33] synthesized silicon modified αTCP using the wet chemical precipitation and solid-state methods, and, in both cases, an additional silicocarnotite phase emerged, regardless of the silicon content in the material. The αTCP-silicocarnotite sub-system was also obtained and studied by Martinez et al. [34]. In our study, the applied wet chemical method allowed for the successful modification of α-tricalcium phosphate with 0.26 wt.% of silicon, as no other crystalline phases containing silicon were found.

The XRD analysis performed for non-modified and AuNPs-modified hybrid granules revealed the presence of broad diffraction peaks profiles, characteristic of a non-stoichiometric apatitic structure with a low crystallinity degree (Figure 1c,d). Polymers do not exhibit long-range structural order, and therefore the diffraction peaks from the chitosan were not visible. Only the amorphous halo from the chitosan was noticed at the diffraction pattern. These results stay in agreement with the findings of our previous studies on hybrid hydroxyapatite/chitosan-based granules [20]. In the case of biomicroconcretes, the XRD measurements demonstrated that composites consisted of two major crystalline phases, namely αTCP (54–62 wt.%) and HA (38–46 wt.%) (Table 2). No other crystalline phases were detected.

### 4.2. Setting Times

According to ISO/DIS 18531 for calcium phosphate-based bone cements, the setting time is the time “required from the start of powdered agent and liquid agent blending until hardening of the cement” [35]. The results of the initial and final setting time measurements with a Gilmore needle apparatus are collected in Table 3.

The initial setting times of developed biomicroconcretes varied between 5–7 min and the final ones between 10–20 min, depending on the solid phase composition. Biomicroconcretes containing granules with AuNPs had shorter setting times in comparison with the control material (HT). Additionally, the modification of αTCP with silicon influences the setting process. The shortest setting times, both initial and final, were noticed for materials containing Si-αTCP as a setting reagent (Au, Si-HT). It has been already shown in the literature that for silicon-modified calcium phosphate cement, some properties are altered compared with its pure counterpart. For example, Wei et al. [36] noticed a higher solubility, whereas Mestres et al. [37] found a faster hydrolysis of Si-αTCP in comparison with non-modified αTCP. As biomicroconcretes’ setting process depends inter alia on the chemistry, specific surface area, and solubility of the setting component, the higher solubility of Si-αTCP can translate into the shorter final setting times recorded for Au, Si-HT. However, these results stay in contradiction to the findings of Mestres et al. [37], who stated that Si-αTCP exhibited a slower setting rate than αTCP. It is possible that hydroxyapatite grains, present in hybrid granules, served as nucleation seeds and influence the kinetic of the setting process of biomicroconcretes.

### 4.3. Compressive Strength

**Figure 2:**
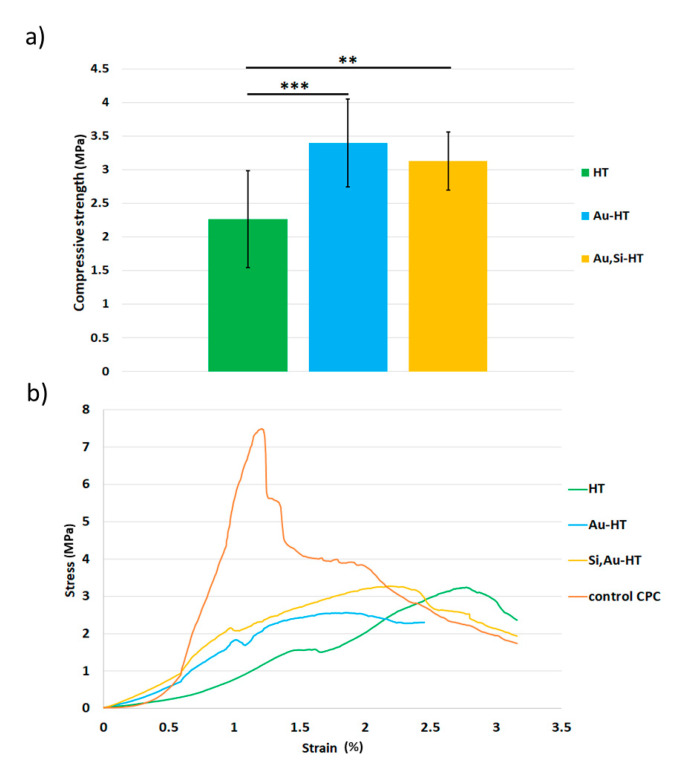
The compressive strength (**a**) and compressive stress–strain curves (**b**) of biomicroconcretes 7 days after setting and hardening. Control CPC-αTCP-based bone cements without granules. Statistically significant differences indicated by ** *p* ≤ 0.01 and *** *p* ≤ 0.001.

The mechanical strength is an important criterion during the selection of bone substitute materials as cracks and the disintegration of biomaterial in the body can lead to acute chronic inflammation. Usually, the main issues influencing the mechanical properties of composites are (I) the kind and amount of coexisting phases, (II) presence or lack of chemical interactions between the constituents, (III) good or bad adhesion of the components, and (IV) homogeneity of material’s microstructure. In our studies, the biomicroconcretes varied in their compressive strength, depending on the type of granules (Figure 2). The results of the compressive tests revealed that the presence of hybrid granules modified with gold nanoparticles positively affected the compressive strength of the composites. In comparison with the material HT (2.3 ± 0.7 MPa), materials Au-HT (3.1 ± 0.4 MPa), and Au, Si-HT (3.4 ± 0.7 MPa) were characterized by a higher mechanical strength. This may also indicate that the intrinsic properties of the granules influenced the mechanical properties of the composites. A one-way ANOVA followed by a post-hoc Tukey HSD test indicated that the observed differences between the materials were statistically significant. Compressive stress-strain curves (Figure 2b) showed that the fracture mechanism of the obtained biomicroconcretes varied from αTCP cement without granules (control CPC). The αTCP material showed a typical brittle behavior, whereas the biomicroconcretes presented rather a composite-type characteristic, where the granules suppressed or stopped crack propagation. The compressive strength of developed biomicroconcretes is lower in comparison with αTCP-based bone cements and biomicroconcretes studied in our previous research [7,8,38]. However, the obtained values fell within a range of values for the compressive strength of cancellous bone (2–12 MPa), and therefore may be sufficient for non-load bearing applications.

### 4.4. Microstructure

**Figure 3:**
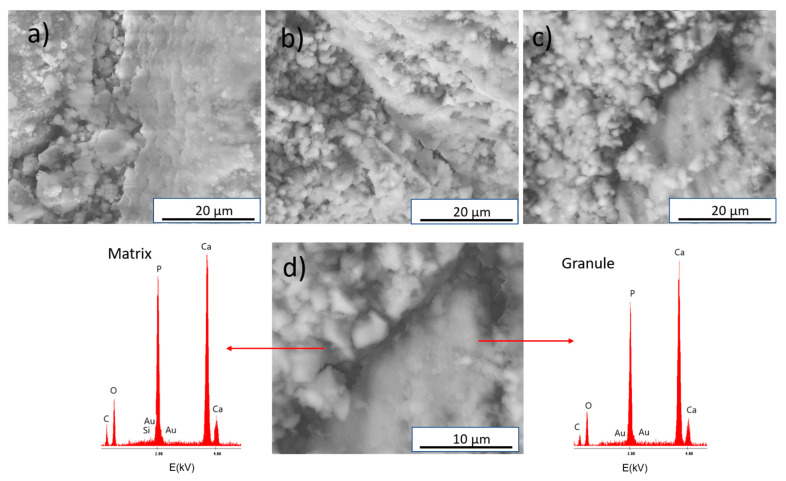
SEM microphotographs of the cross-sections of materials: (**a**) HT, (**b**) Au-HT, (**c**) Au, Si-HT, and (**d**) results of EDS analysis of Au, Si-HT.

The results of the SEM (Scanning Electron Microscopy) and TEM(Transmission Electron Microscopy) observations demonstrated that the obtained materials were characterized by a homogeneous microstructure for AuNPs and silicon modified and non-modified biomicroconcretes. The SEM observations of the composites revealed the presence of hybrid granules embedded in a microporous matrix composed of CaPs grains (Figure 3). EDS analysis in the microareas confirmed the presence of gold for Au-HT (result not shown), as well as gold and silicon for Au, Si-HT (Figure 3d, Figure S1—Supplementary Material).

**Figure 4:**
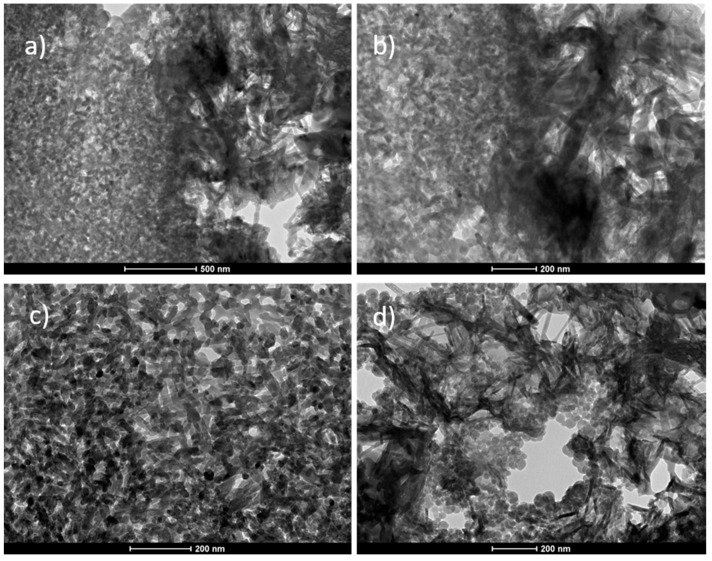
TEM/BF microphotographs of the HT composite presenting: matrix/granule interface (**a**,**b**), and the microstructure of the hybrid granule (**c**) and calcium-phosphate matrix (**d**).

The TEM studies revealed that the matrix of the biomicroconcretes displayed a microporous microstructure consisting of entangled needle-like and round-to-ovoid apatitic crystals (Figure 4d). The microstructure of the hybrid granules was more compact and consisted of nanometric apatite grains (Figure 4c). The SEM and TEM images of materials showed that components were well-combined. In the case of all of the studied materials, a good adhesion between CaPs grains and hybrid granules at the matrix/granule interface was observed.

### 4.5. Chemical Stability and In Vitro Bioactivity

**Figure 5:**
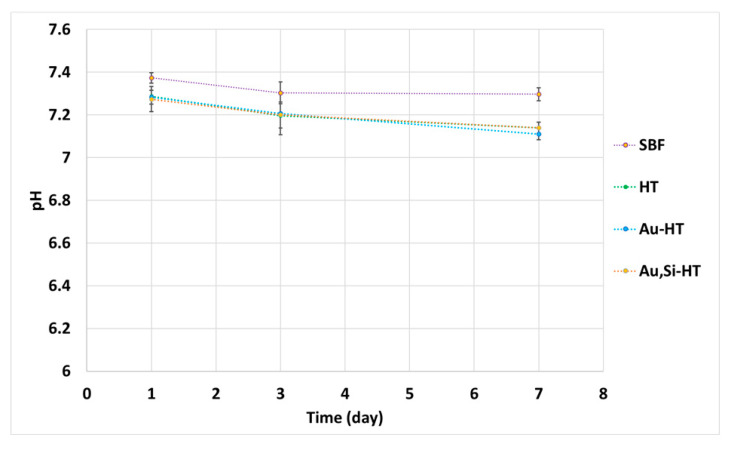
pH versus biomicroconcretes’ incubation time in simulated body fluid.

The chemical stability tests showed that during the 7-day incubation, a slight decrease in pH of SBF from 7.40 to 7.15 was noticed (Figure 5). Nevertheless, the registered changes of pH in SBF around the biomicroconcrete samples remained close to the physiological one.

**Figure 6:**
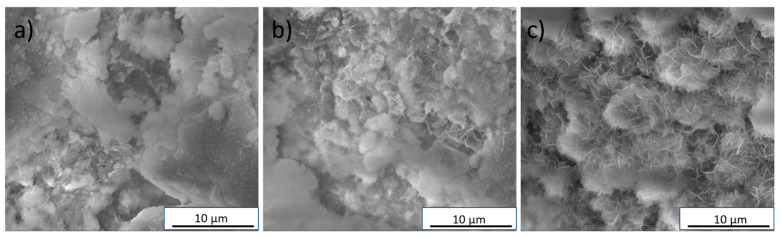
SEM microphotographs of the following materials: (**a**) HT, (**b**) Au-HT, and (**c**) Au, Si-HT after 7 days of incubation in SBF.

Immersion experiments are considered to allow for assessing the bioactive potential of biomaterials. The presence of an apatite layer after incubation in simulated body fluid may indicate that the tested material has a bioactive potential and after implantation will form a chemical bonding with the bone tissue [31]. In this study, after 7 days of incubation in SBF, an apatite layer was observed on all of the biomicroconcretes’ surfaces (Figure 6). However, the material Au, Si-HT seemed to possess the largest amount of cauliflower-like apatite agglomerates, which may be connected with a faster dissolution process of the Si-αTCP matrix in comparison with αTCP, and may indicate its higher bioactive potential.

### 4.6. In Vitro Antibacterial Activity

**Figure 7:**
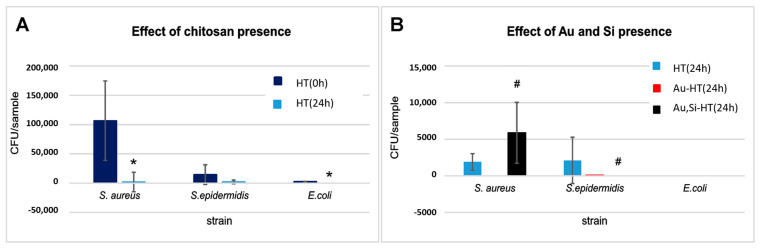
Effect of chitosan presence in the material (**A**) and effect of Si and Au presence in biomicroconcretes (**B**) observed after 24 h incubation with 3 bacterial strains. * significance compared to control at 0 h (Mann–Whitney test); # significance compared to control at 24 h (one-way ANOVA followed by Dunnett’s post hoc test). CFU: colony-forming unit.

The results of the in vitro antibacterial activity measurements are presented in Figure 7.

As shown in Figure 7A, the amounts of bacteria recovered from the control HT material immediately after inoculation (0 h) differed significantly depending on the strain used in the particular test. This was observed despite the fact that the initial quantity of bacterial cells introduced into the samples was similar in the case of all of the bacteria. Therefore, the material HT showed a notable ability to adsorb *S. epidermidis* and *E. coli* cells, in comparison with *S. aureus* bacterial cells. After 24 h of incubation, biomicroconcrete caused a significant reduction of recovered bacteria (for *E. coli*, no bacterial cell was recovered). Material HT itself likely shows a high antibacterial potential, probably due to the presence of chitosan. This effect of chitosan is widely known. Although intensely studied for the last decades, the mechanism of the antibacterial action of chitosan is not completely clear. The most acceptable one presumes the appearance of an interaction between positively charged chitosan molecules (electrostatic forces between the protonated NH_3_^+^ groups) and negatively charged microbial cell membranes, possibly by competing with Ca^2+^ for electronegative sites on the membrane surface [39,40]. It was therefore not surprising that chitosan-enriched cements, incubated with bacterial inoculates, caused a significant reduction in the viability of bacteria.

Figure 7B shows the comparison of the number of bacteria recovered after 24 h of incubation from AuNPs and AuNPs/Si-modified biomicroconcretes and control material (HT). For the *S. epidermidis* strain, the bacteria-killing effect of AuNPs and, in particular of AuNPs/Si-modification, is clear. For *S. aureus*, only Au-HT cement killed the bacteria completely, while the Au, Si-HT material surprisingly caused an increase in the number of surviving bacteria (Figure 7B). In the literature, the antibacterial effect of silicon is well known and documented. However, some studies show no antibacterial activity or even a growth accelerating effect of silicon on *Staphylococcus aureus* [41,42]. Thus, a slight increase in the number of *S. aureus* cells surviving the contact with the Si/Au-enriched sample observed in comparison with HT and Au-HT materials may be a consequence of both combined phenomena: bactericidal effect of Au and growth stimulating effect of Si. Further investigations are needed to explain the phenomenon. The effect of AuNPs and AuNPs/Si-modification on *E. coli* viability was impossible evaluate because no bacteria were recovered from the HT control.

For better clarity, the percent of reduction of bacterial viability after 24 h of incubation with the tested samples was calculated (Table 4). It was found that the percent reduction of viable bacteria by Au-HT and Au, Si-HT composites varied within the range 94.5–100%. This confirmed the high antibacterial potential of AuNPs and Si-modified materials. However, the percent viability of the tested bacteria for the control non-modified HT biomicroconcrete observed after 24 h of incubation was also high: 98.2–100% for the *S. aureus* and *E. coli* strains, and slightly lower (85.6%) for the *S. epidermidis* strain (Table 4).

The effect of the gold nanoparticles and silicon introduced into the samples was difficult to estimate because of the high impact of chitosan’s presence on bacterial survivability, in particular for the *S. aureus* and *E. coli* strains. However, for the *S. epidermidis* strain, it was noted that the percent reduction of bacteria increased after 24 h incubation from 85.6% for the control sample to 99.4–100% for the AuNPs and Si-modified biomicroconcretes (Table 4). Therefore, this may suggest the possible beneficial impact of AuNPs and Si by enhancing the antibacterial activity of chitosan. These results stay in agreement with the findings of Kurtjak et al. [19], who stated that Au nanoparticles reveal an effective antibacterial action against *E. coli* and *S. epidermidis*. The antibacterial activity against *E. coli* was also reported by Cui et al. [43]. Generally, the major processes underlying the antibacterial effects of nanoparticles are the (I) disruption of the bacterial cell membrane, (II) generation of reactive oxygen species (ROS), (III) penetration of the bacterial cell membrane, and (IV) induction of intracellular antibacterial effects (e.g., interactions with DNA and proteins) [22]. Because AuNPs are relatively inert, they do not exhibit an apparent intrinsic antibacterial activity. It is believed that their main mechanism of bacterial toxicity is related to the direct adhesion of AuNPs onto the bacterial surface [21]. This mechanism is highly dependent on the nanoparticle size, and typically, smaller nanoparticles exhibit lower minimal inhibitory concentrations (MIC). However, because of the low reactivity, AuNPs need to be used in higher concentrations to produce the same bactericidal effect as other metal-based nanoparticles [22,44]. This may be the reason the antibacterial action of AuNPs in the presence of chitosan was not clearly visible. The effect of gold nanoparticles on the antibacterial properties of biomicroconcretes requires further research. Moreover, although the three bacterial strains selected for this study are claimed to be the most common in bone implants infections [45,46], the response of other bacterial strains to the tested microconcretes may vary in a significant way. Therefore, the antibacterial potential of Au-HT and Au, Si-HT should be verified individually for each bacterial strain of particular interest.

## 5. Conclusions

In this study, we developed surgically handy biomicroconcretes on the basis of αTCP or Si-αTCP (matrix) and hybrid hydroxyapatite/chitosan-based granules non-modified and modified with gold nanoparticles (AuNPs). Such composites combined the dual functions of the antibacterial action and bone defect repair.

The results of our studies revealed that modification with AuNPs and silicon influenced both the setting time and compressive strength of the composites. The addition of AuNPs decreased the final setting time from 20 min (HT) to 16 min (Au-HT). Further modification with silicon reduced the final setting time of the materials to 10 min. It has been also shown that the introduction of gold nanoparticles slightly increased the compressive strength of biomicroconcretes from 2.3 ± 0.7 MPa (HT) to 3.4 ± 0.7 MPa (Au-HT). The strengthening effect may be explained by the intrinsic properties of AuNPs-modified hybrid granules. The obtained compressive strength of the biomicroconcretes was sufficient for non-load bearing applications. SEM and TEM observations showed that all of the developed biomicroconcretes possessed a homogenous microstructure characterized by the good adhesion between CaPs grains and hybrid granules at the matrix/granule interface.

All of the studied composites possessed a bioactive potential, proven in in vitro studies in simulated body fluid. The material modified simultaneously with AuNPs and silicon (Au, Si-HT) possessed the largest amount of cauliflower-like apatite agglomerates on its surface after 7 days of incubation in SBF. This is probably due to the more rapid dissolution of the Si-αTCP matrix in comparison with silicon-free counterparts. The faster development of the apatite layer indicated a higher bioactive potential of silicon-modified materials.

The results of our studies confirmed the antibacterial properties of the developed inorganic-organic type biomicroconcretes. This was due to the presence of chitosan, characterized by high antibacterial and fungicidal activities, as well as the addition of gold nanoparticles. The strongest effect on the bacterial viability was noticed in the case of AuNPs and Si-modified biomicroconcretes. The bactericidal action was particularly visible for the *S. epidermidis* strain. The exact antibacterial effect of the modifiers was difficult to estimate because of the high parallel influence of chitosan on the bacterial survivability. Nevertheless, the possible beneficial impact of AuNPs and silicon, enhancing the antibacterial activity of chitosan, was stated, and further tests are needed to confirm this.

## Figures and Tables

**Table 1:** Compositions of the studied materials.

Material	Solid Phase		Liquid Phase	L/P (g/g)
	Granules (40 wt.%)	Powder (60 wt.%)		
HT	HA/CTS	αTCP	0.75 wt.% methylcellulose in 2.0 wt.% Na_2_HPO_4_	0.6
Au-HT	Au-HA/CST	αTCP		
Au, Si-HT	Au-HA/CST	Si-αTCP		

HT (letters from: H-hybrid HA/CTS granules and T-αTCP powder); CTS (letters from: ChiToSan); HA (letters from: HydroxyApatite).

**Table 2:** Phase compositions of the initial powders, granules and set biomicroconcretes.

Material		αTCP (wt.%)	HA (wt.%)
Powders	αTCP initial	98 ± 2	2 ± 2
	Si-αTCP initial	97 ± 1	3 ± 1
Granules	HAp/CTS	-	100 ± 0
	Au-HAp/CTS	-	100 ± 0
Biomicroconcretes	HT	54 ± 4	46 ± 4
	Au-HT	62 ± 2	38 ± 2
	Au, Si-HT	62 ± 2	38 ± 2

**Table 3:** The setting times of biomicroconcretes.

Material	Setting Time (min)	
	Initial	Final
HT	7 ± 1	20 ± 1
Au-HT	6 ± 1	16 ± 1
Au, Si-HT	5 ± 1	10 ± 1

**Table 4:** Percentage reduction of bacterial viability after incubation for 24 h with the tested cements.

Strain	Material		
	HT	Au-HT	Au, Si-HT
*S. aureus*	98.2	100	94.5
*S. epidermidis*	85.6	99.4	100
*E. coli*	100	100	100

## Data Availability

All the data is available within the manuscript.

## Supplementary Materials

The following are available online at https://www.mdpi.com/article/10.3390/ma14143854/s1, Figure S1: The scanning electron microscope (SEM) image and the elemental mapping of the surface of Au,Si-HT material.
