# Supplementary material for: Effect of Gold Nanoparticles and Silicon on the Bioactivity and Antibacterial Properties of Hydroxyapatite/Chitosan/Tricalcium Phosphate-Based Biomicroconcretes
Source: Materials (Basel). 2021 Jul 9;14(14):3854. doi: 10.3390/ma14143854 (PMC8304576; doi:10.3390/ma14143854)
Supplement: Supplementary file 1 [file materials-14-03854-s001.zip › materials-1272423-supplementary.pdf]

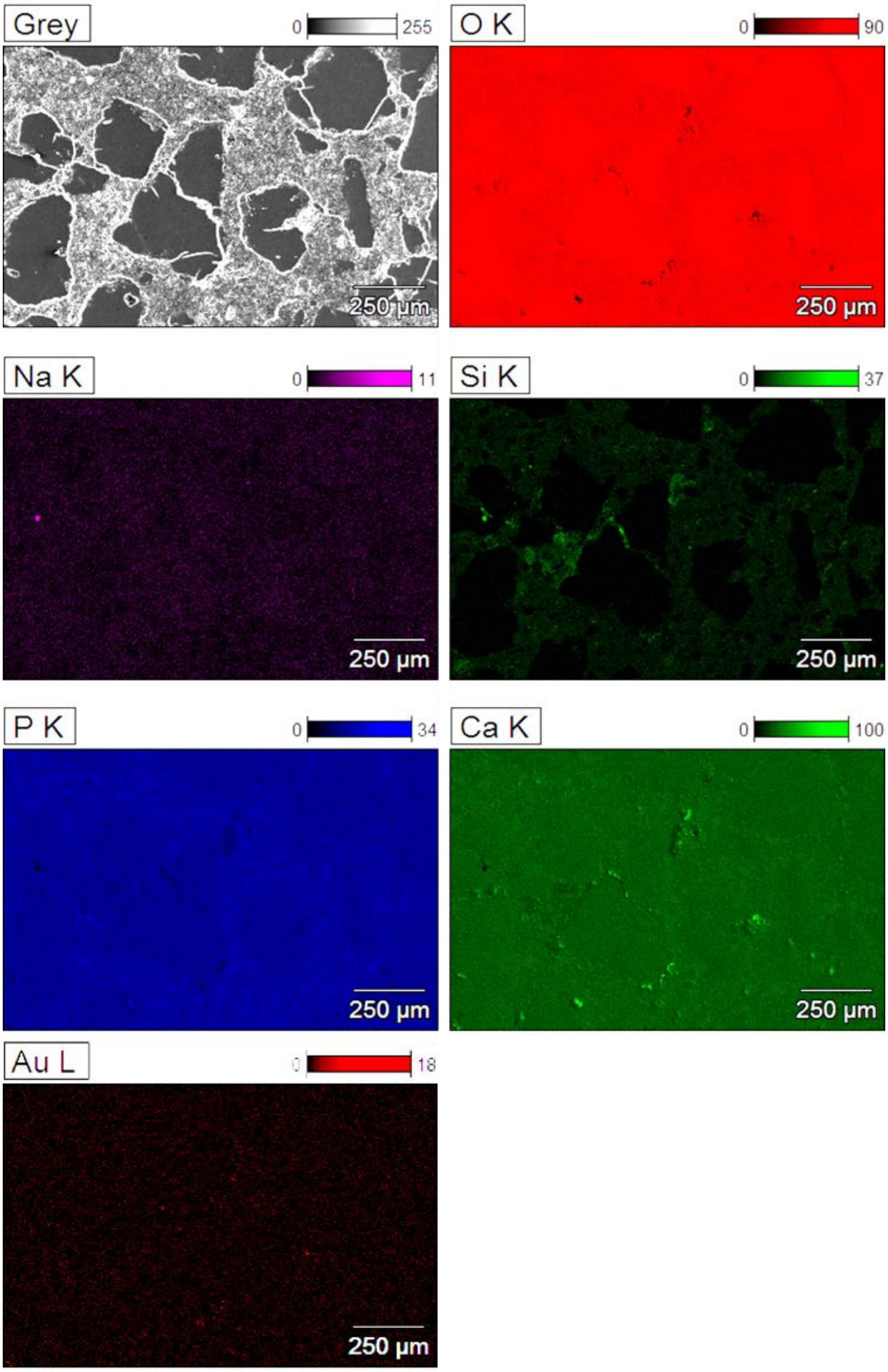

Figure S1. The scanning electron microscope (SEM) image and the elemental mapping of the surface of Au,Si-HT material.
